# Advancing research on strategies to reduce drug use and overdose-related harms: a community informed approach to establishing common data elements

**DOI:** 10.1186/s12954-025-01301-0

**Published:** 2025-10-15

**Authors:** Lissette M. Saavedra, Mia C. Christopher, Dora Illei, Alex H. Kral, Bradley Ray, Jon E. Zibbell, Karla D. Wagner, Annick Borquez, Ayana Jordan, David Seal, Magdalena Cerdá, Mary Ellen Mackesy-Amiti, J. Deanna Wilson, Mai T. Pho, Czarina Navos Behrends, Hira Hassan, Catherine Tomko, Emmanuel Oga, Jessica D. Cance

**Affiliations:** 1 RTI International, Research Triangle Park, NC USA; 2 Department of Health Behavior, Policy, and Administration Sciences, School of Public Health, University of Nevada, Reno, NV USA; 3 School of Medicine, University of California San Diego, San Diego, CA USA; 4 New York University Grossman School of Medicine, New York, USA; 5 Tulane University School of Public Health and Tropical Medicine, New Orleans, LA USA; 6 Department of Population Health, New York University Grossman School of Medicine, New York, USA; 7 School of Public Health, University of Illinois Chicago, Chicago, IL USA; 8 Department of Family Medicine and Community Health, University of Pennsylvania, Philadelphia, PA USA; 9 Biological Sciences Division, University of Chicago, Chicago, IL USA; 10 Department of Population Health Science, Weill Cornell Medical College, New York, NY USA; 11 Department of Mental Health, Johns Hopkins Bloomberg School of Public Health, Baltimore, MD USA

**Keywords:** Common data elements, Common outcome measures, Community informed, Harm reduction, Overdose, Delphi

## Abstract

With the overdose crisis continuing to pose significant challenges in North America, harm reduction strategies are critical for public health systems to reduce mortality and morbidity. Despite the considerable strides in harm reduction research, high-quality evidence for decision-making is limited. This is compounded by a variation in reported outcomes, drug supply, administration changes, and policy and social impacts, which further challenge researchers and practitioners in their efforts to implement effective, nimble harm reduction interventions. Adoption of common data elements (CDEs) and common outcome measures (COMs) helps researchers standardize and enhance data collection and outcome reporting, ultimately improving the comparability and generalizability of research findings. To accelerate the pace and use of CDEs, members of the NIDA HEAL Research on Interventions for Stability and Engagement (RISE) engaged in prospective semantic harmonization and consensus on CDEs and COMs using a rigorous pragmatic Delphi community informed approach. This process resulted in a set of CDEs and COMs that standardized data collection and reporting across 10 harm reduction research projects. This paper describes this process and presents the derived CDEs and COMs, along with key considerations, challenges encountered, and lessons learned.

With the overdose crisis continuing to pose significant challenges in North America, harm reduction strategies are critical for public health systems to deliver optimal evidence-based prevention and care [1, 2]. Despite the considerable strides in harm reduction research, high-quality evidence for decision-makers is often limited [3, 4]. This is compounded by the inconsistent use of validated harm reduction assessment tools and variation of instruments across studies, which make it difficult to interpret and generalize findings, archive data for future use, and harness the power of cumulative science [5, 6]. Moreover, the variation in reported outcomes, such as substance use and routes of administration, as well as varied policy and social impacts, further challenge researchers and practitioners’ efforts to implement effective harm reduction interventions. High quality evidence is not only essential for policymakers and researchers but also for the people who use drugs and the programs that serve them. Community organizations, peer networks, and frontline implementers rely on strong and consistent data (from research and practice) to guide practice, improve services and advocate for policies grounded in real world outcomes.

The absence of standardized practices in research extends beyond harm reduction and has been observed across various disciplines [2]. Common data elements (CDEs) are standardized questions and items used to consistently collect data across different research studies [7]. These elements uphold the National Institutes of Health’s (NIH) FAIR principle, ensuring research data is *findable*,* accessible*,* interoperable*, and *reusable* [8, 9]. Regulatory agencies and funders have increasingly emphasized the need to standardize both the execution and reporting of clinical trials and research studies in general, and embracing CDEs and standardized definitions in harm reduction research can advance and accelerate collaboration; enhance the consistency and quality of data collection; streamline study setup; and facilitate cross-study comparisons, meta-analyses, and data integration [10–13]. Equally important, people who use drugs and those working within harm reduction programs rely on evidence and practice informed programming to ensure services are relevant, responsive and rooted in real world experience. Standardized data supports the identification, refinement, and evaluation of programs that are directly aligned with the needs and goals of those most affected by the overdose crisis [14].

In the dynamic field of harm reduction, the establishment of CDEs is essential for advancing research, informing policy, and guiding practical applications. Benefits of researcher uptake and ongoing updates of the RISE CDEs are many and key to the rapidly changing landscape of harm reduction. These benefits include facilitating standardization and comparability across studies. By employing consistent data collection methods, researchers can accurately compare results and perform comprehensive meta-analyses [15]. This standardization reduces variability and enhances the reliability of findings, contributing to a more robust evidence base. Additionally, the quality of data is significantly improved because standardized protocols minimize errors and biases that might otherwise compromise study outcomes. With standardized data elements, data sharing is enhanced, fostering a collaborative environment that supports larger and more diverse datasets [16]. Collaboration extends to the design and implementation of research projects, where the use of predefined data elements and outcome measures expedites these processes, allowing researchers a base for data capture consideration.

In this paper, we describe the HEAL Research on Interventions for Stability and Engagement (RISE) Network community-informed approach for selection of flexible CDEs through expert consensus. The RISE is part of the NIH Helping End Addiction Long-Term^®^ (HEAL) initiative and includes 10 research projects that are described in Table 1. The paper also discusses the process and challenges in developing the RISE CDEs, and an overlap mapping of data being collected from RISE projects with the standardized instrument.

## Methods

The 10 research projects are led by members the RISE Network, who make up a national Network of investigators that aim to study and improve the effectiveness, implementation, and impact of existing and new harm reduction policies and practices [18]. Research supported by these programs was conducted in real-world settings and in collaboration with a diverse range of community partners including people with lived experience, who help make the strategies being studied sustainable and scalable. The RISE Network Coordinating Center supports the research projects by streamlining communication across the Network, providing support related to data methodology, engaging community stakeholders in all Network activities, and translating research findings so they can be used by researchers, practitioners, and communities. The 10 studies involved in this initiative were selected based on their relevance to harm reduction strategies and their potential to contribute valuable data. Each study focuses on harm reduction strategies including syringe service programs (SSPs), and in some instances other substance use treatment strategies including medications for opioid use disorder, contingency management and overdose prevention strategies. Table 1 includes a description of each research project and their study focus.

**Table 1:** Participating research projects in the RISE and interventions implemented

Project Title	Institution	Population	Location	Intervention
Implementing and Evaluating the Impact of Novel Mobile Harm Reduction Services on Overdose Among Women Who Use Drugs (SHOUT)	Johns Hopkins University	Women who use drugs	Baltimore, MD	Mobile SPARC services, a novel mobile harm reduction service to reduce overdose among women who use drugs
Culturally Responsive Integrated Harm Reduction Services for Black and Latinx People Who use Drugs	New York University (NYU)	Black and Latinx people who use drugs	Bronx, NY, and New Haven, CT	8-week culturally responsive, integrated harm reduction services
Peers Expanding Engagement in Methamphetamine Harm-Reduction with Contingency Management (PEER-CM)	Oregon Health and Science University	People using methamphetamine	Oregon	A project that uses incentives to achieve self-identified, personal harm reduction goals to increase engagement with harm reduction and treatment services and reduce the likelihood of overdose among people using methamphetamine
Assessing the Reach, Effectiveness, and Implementation of Multiple Harm Reduction Interventions	RTI International	People who inject or smoke illicit drugs	San Francisco, CA	Multiple harm reduction interventions
Teaching Harm Reduction in Vulnerable Environments (THRIVE)	University of Pennsylvania	People who use drugs	Pittsburgh, PA	A peer-led intervention using a human-centered design approach
Mobile Health Strategies to Support Longitudinal Engagement in Comprehensive, Community-based Prevention Services for People Who Use Drugs	University of Wisconsin-Madison and Tulane University	People who use opioid and/or stimulant drugs	Wisconsin	Mobile health strategies for harm reduction
Promoting Remote Harm Reduction and Secondary Services in Rural Settings (PROMOTE)	University of Chicago Medicine and University of Illinois at Chicago	People who use drugs	Illinois	Secondary distribution of harm reduction supplies including sterile syringes, HIV and hepatitis C virus self-test kits, fentanyl test strips, and naloxone, and a remote toolkit
A Network-based, Mixed Methods Study to Identify and Support Multiple Overdose Responders and Inform Overdose Prevention Interventions	University of Nevada, Reno	Overdose responders	Reno, NV	Support for multiple overdose responders
Expansion of Mail-Delivered Harm Reduction Services in the U.S.	Weill Cornell Medical College	Mail delivery clients	National Survey	Mail-delivered harm reduction services
Comparative Evaluation of Overdose Prevention Centers in New York and Rhode Island(SAFER Study)	NYU and Brown University (NYU-Brown)	Communities with overdose prevention centers	New York City and Rhode Island	Overdose prevention centers

To ensure a bidirectional process, the RISE Coordination Center used a Delphi method to reach consensus [13]. The Delphi model is a methodological approach that leverages expert input via iterative rounds of questionnaires, and which is often employed in the development of CDEs. The Delphi process includes several key steps, such as the generation of a list of data elements and constructs, formation of a Delphi expert panel (in this case, RISE Networkdata harmonization workgroup members and project-affiliated community partners including people with lived experience), multiple rounds of voting on data elements, analysis of results, and dissemination of the summarized results for further voting and feedback. The process continues until reaching consensus [17]. Table 2 outlines each iteration.

**Table 2:** Systematic iteration process for tier 1 measures

Iteration	Description	Content Reviewed	Rating System	Decision
1	Distribution of the initial compilation of measures among research sites, community partners, subject matter experts, persons with lived experience, and racial, gender, and sexual minorities. Coordinating Center refines the next compilation from scores and feedback.	Measure -level; Tiers 1–3	0 = reject; 1 = reject/revisit with significant revisions; 2 = accept with minor edits; 3 = accept as is	Measures eliminated are no longer considered for Tiers 1–3
2	Distribution of the refined compilation of measures. Similar measures are grouped together under constructs; sites are encouraged to keep one measure per grouping. Coordinating Center refines the next compilation.	Measure -level; Tiers 1–3	“Keep for consideration” or “Reject”	Measures accepted were moved to Tier 1, measures rejected moved to Tiers 2 or 3
3	Consideration of a refined list of measures for inclusion in Tier 1.	Measure -level; Tiers 1–3	Qualitative feedback	Measures accepted were moved to Tier 1, measures rejected moved to Tiers 2 or 3
4	Update of Tier 1 measures based on qualitative comments from Iteration 3.	Measure - level; Tier 1	No rating system	Internal edits made based on RISE internal comments
5	Compilation of measures with conflicting feedback or lack of consensus as well as the feedback. Distribution to sites for evaluation.	Measure level; Tier 1	Yes/No	Measures with clear outcomes were moved to Tier 1, measures without consensus were labeled “Tier 1 under review”
6	Scrutiny of “under review” Tier 1 measures.	Measure level; Tier 1	Virtual meeting with live qualitative feedback and discussion	Detailed edits addressed during virtual call, edits to each Tier 1 measure finalized
7	Compilation of edited and approved Tier 1 measures and their distribution to the research projects for review (including a newly joined project, NYU-Brown).	Measure level, Tier 1	Qualitative – from new project only	Measures where feedback warrants further discussion with full network are compiled
8	Measures from Tier 1 discussed based on new project feedback, categorized, and finalized.	Measure level; Tier 1	Virtual meeting with live qualitative feedback and discussion	Any proposed changes discussed and consensus reached among the research sites; Tier 1 measures are finalized

### Community partner inputs

It is important for RISE Networkresearchers, who aim to understand the contextual rationales behind the decisions made by other Network members, in addition to their own, to find common ground as a collaborative Network. Principal investigators (PI) and other community experts which included people who used drugs, from 10 research projects served as the Delphi expert panel. Each research project PI had voting power on behalf of their project, unless they specifically designated other team members to vote on behalf of the research project. It is important to note that between iterations, representatives shared items under consideration with their teams and the individual study. Community Advisory Boards often brought back important comments, feedback and modifications. These were incorporated in several iterations. For Iterations 1 and 2, research projects submitted their votes and feedback to the Coordinating center independently, unaware of how other research projects voted. However, in each subsequent iteration, project-level voting and feedback was documented in the distributed results so projects can learn from insights shared by community partners. Secondly, the RISE Network expert panelists were part of a Network of grantees awarded by NIH (RISE), and thus were not unrelated reviewers. Lastly, although research project team members were reviewing measures for inclusion as CDEs, they also considered their research project and protocol and made sure their decisions aligned with broad applicability and contribution to the harm reduction field in addition to individual research projects.

### The need for a systematic iterative approach

Capturing prospective CDEs requires focused and continuous inputs from partners and key informants, especially people who use drugs and providers who serve them. This is especially true working in the ever-evolving harm reduction space. The RISE Network Coordinating Center carefully considered the unique needs of each research site, factoring in, among other criteria, geographic location and specific research objectives. Policy, local drug markets, and local vernacular played a role in the types of data each research project could collect. For example, some research projects did not have access to SSPs, limiting their ability to add SSP measures to their survey instruments, whereas other research projects had main outcomes related to SSPs. Terminology used by people who use drugs varied by research project location. For example, one research project referred to methamphetamine in combination with heroin as a “goofball,” a term that at the time some other research projects were not as familiar with. This difference in vernacular exemplified the importance of tailoring CDEs to local context. Thus, the CDE remained consistent across, and variants appear in parenthesis allowing for research project tailoring. Research objectives also differed significantly across research projects; this influenced what measures each research team was willing to collect. Each research project needed to balance prioritizing their specific research questions, population, and external constraints (for example, one project was only able to use available local electronic health records), while also recognizing the potential contribution of CDEs to the broader harm reduction field and maximizing RISE’s impact.

Multiple iterations of review and feedback were useful for reaching consensus. In the Iterations 1 and 2, research projects voted on and provided measure-level qualitative feedback independently. However, as the iteration continued, the feedback became increasingly nuanced and varied across research projects. This made it crucial, starting in Iteration 3, for research projects to observe the voting and input of other research projects in the RISE Network to understand the distinct requirements and needs of the other research projects. As the RISE network progressed through each iteration, measures were either eliminated or moved to other tiers. Adaptations to measures, informed by qualitative feedback, were further incorporated or expanded upon with each subsequent iteration. When consensus was not easily reached on specific measures through open ratings and feedback, RISE network members convened in virtual meetings to address the specifics and find a resolution that was acceptable to each research project. Through this process, measures were collectively refined and tailored to the preferences and feasibility (timescale recall considerations, e.g., 30 days vs. 7 days) of the entire group.

### Development of CDEs using semantic methodology and a systematic iterative approach to define core domains and constructs

**Fig. 1:**
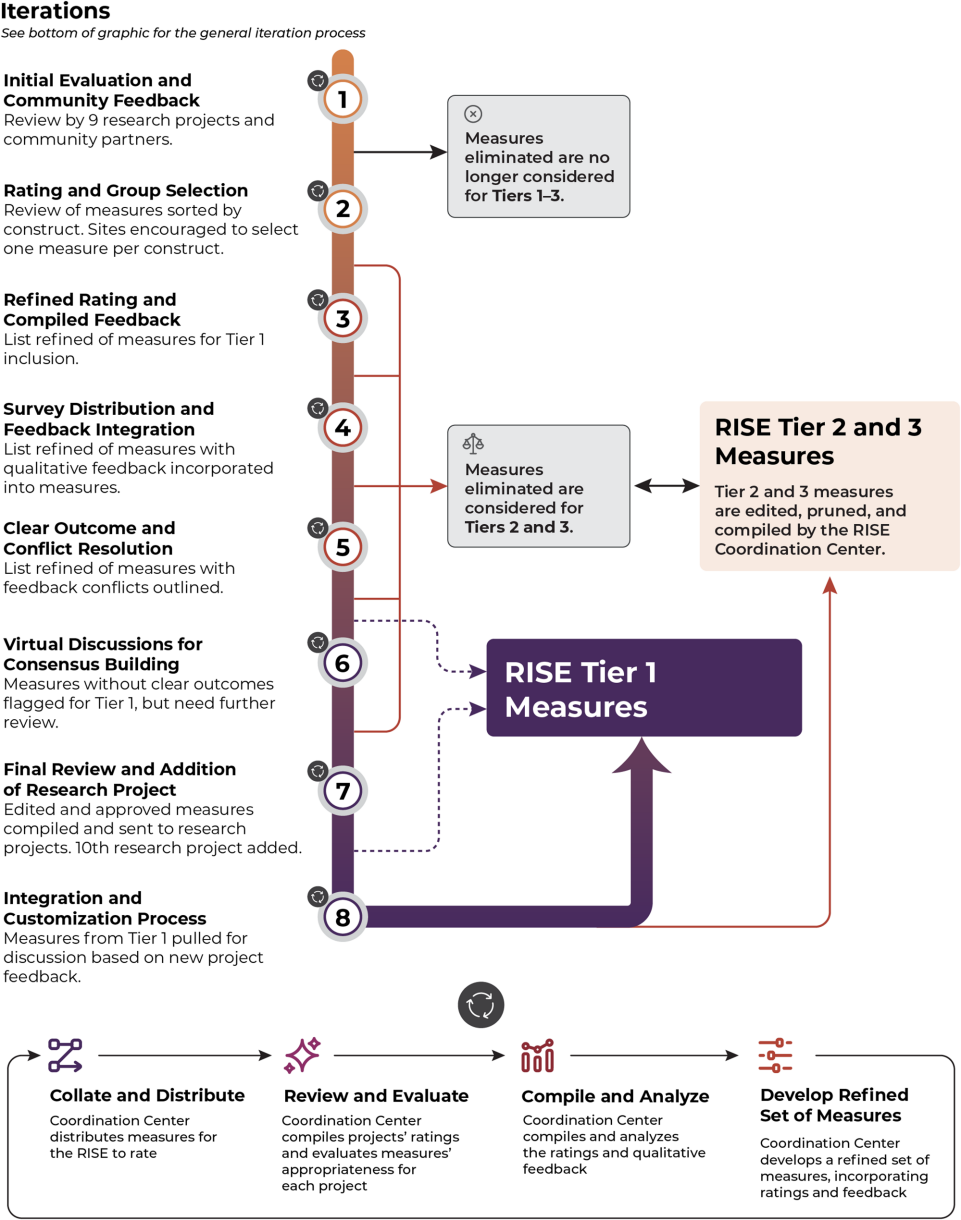
RISE Iteration Cycle Details

The RISE Network first identified priority measure constructs to include in the CDEs’ data harmonization process during an in-person meeting. Research projects then submitted their draft survey instruments to the Coordination Center. Research project principal investigators were given the opportunity to share additional validated instruments to include in the RISE Network data harmonization process. The Coordination Center organized measures by the predetermined constructs and included measures from previously validated instruments for the voting consensus process. Previously validated instruments included the NIH HEAL Initiative’s CDEs [18]. Almost all iterations included some version of 1). collating and distributing measures for review to research projects, 2). research site review and evaluation of measures, 3). compiling and analysis feedback (qualitative and quantitative), and 4). developing a refined set of measures to distribute in the next iteration until consensus is reached as illustrated in Fig. 1. To guide selection of candidate CDEs while maintaining flexibility across the diverse research projects, the RISE Networkadopted a 3-tier system for classifying common data elements: Tier 1 CDEs were core elements endorsed across all projects and considered essential for cross-project comparison. Tier 2 CDEs were strongly supported but not universally applicable, and Tier 3 CDEs were site-specific or exploratory measures that held promise for future standardization. This tiered approach allowed for both standardization and local adaptation. Also, where possible, existing instruments including those developed for the NIH HEAL CDE initiatives were used as source materials for candidate measures. Some were adopted in full, while other required adaptation to align with the specific needs of harm reduction populations or to reduce response burden. Adaptations were tracked carefully to preserve the intent of the original constructs while improving feasibility across projects. Although modifications may limit comparability to studies using original versions, the Network prioritized measures that could be feasibly and meaningfully implemented in harm reduction contexts.

### Systematic iteration overview

In the development of Tier 1 measures, each iteration included collection and evaluation of measures followed by analysis for development of a further refined set of measures. Table 2; Fig. 1 provide an overview of each iteration.

*Iteration 1. Initial Evaluation and Community Partner Feedback*. The RISE distributed the compilation of measures (organized by construct) to each of the research projects for evaluation (to consider for inclusion in their survey instrument) and encouraged teams to gather input through their established networks of community partners. These included people who use drugs and other relevant lived experience, community advisory boards, and peer-led groups. While consultation methods varied across sites, all research projects were required to incorporate community feedback as part of their funded protocols, and this feedback played a foundational role in the evaluation of candidate measures. Research projects rated each measure on a scale of 0–3. Zero indicated reject measure, one indicated reject or revisit with significant revisions, two indicated accept with minor edits, and three indicated accepting the measures as is. All projects contributed and all project community partners reviewed. The Coordinating Center analyzed and compiled the ratings and qualitative comments and conducted an internal review using Coordinating Center subject matter expertise. Measures that did not have sufficient consensus at this stage were no longer considered for inclusion as CDEs for RISE (and were not included for Tier 2 or 3). Research projects were separately polled to reassess constructs of interest to ensure that removal of measures did not imply the elimination of the construct.

*Iteration 2. Rating and Group Consensus*. A refined compilation of measures was distributed to research projects for another iteration of rating. Research projects rated each measure as either “Keep for consideration” or “Reject.” Within each construct, similar measures were grouped together and research projects were encouraged to select one from each group. The RISE Network Coordinating Center analyzed rating and qualitative feedback and recorded the number of Research Projects that accepted or rejected each measure. Accepted measures at this stage were considered for the next iteration of voting for Tier 1 measures. Measures that were not included for Tier 1 were considered for Tiers 2 or 3.

*Iteration 3. Refined Rating and Compiled Feedback*. The RISE Network Coordinating Center distributed a refined list of measures for potential inclusion in Tier 1 for rating and qualitative feedback to research projects. The Coordinating Center included previous site scoring and specific qualitative feedback in this iteration. The Coordinating Center compiled the new ratings and tracked the number of research projects that rejected or accepted each measure. Qualitative feedback from each site was compiled by measure. Measures that were accepted at this stage were considered for Tier 1 and measures that were eliminated therefore considered for Tiers 2 or 3.

*Iteration 4. Survey Distribution and Feedback Integration*. Qualitative comments from prior rounds of feedback were incorporated as changes to the new Tier 1 measures. Tier 1 measures were compiled in survey form and distributed to the Research projects with prior comments and ratings. Projects submitted feedback by rating measures and submitting qualitative feedback where needed. The Coordinating Center compiled the feedback.

*Iteration 5. Clear Outcome and Conflict Resolution*. For measures where voting resulted in clear approval, measures were moved to Tier 1 approved list. For measures where research projects had conflicting feedback, measures were compiled, refined, and distributed for a definitive yes/no vote to determine which version or edit would be reflected in Tier 1 measures at times requiring several iterations.

*Iteration 6. Virtual Discussions for Consensus Building*. For the Tier 1 measures under review, virtual meetings were held for further discussion by research projects. Measures and specific edits were discussed, and consensus was reach by voting in virtual meeting.

*Iteration 7. Final Review and Addition of Research Project*. In this iteration, a recently awarded R01 project was introduced. They reviewed Tier 1 measures compiled to date and provided qualitative feedback. Broadly, Tier 1 measures that were approved and voted on were then compiled and distributed for the research projects to review with request for qualitative feedback. This was another opportunity to share with community partners.

*Iteration 8. Integration and Customization Process*. The Coordinating Center evaluated the qualitative feedback from a new research project addition and categorized feedback in four distinct ways as with previous iterations. For the final category of measures, the newly added research project was able to adopt Tier 1 measures without edits.

### Important considerations

Research projects’ buy-in and engagement were essential for achieving consensus. While significant consensus could be achieved through independent, individual feedback submitted online, virtual live meetings were also necessary. Throughout the process, the RISE Network held one-hour meetings bimonthly, although there were instances that necessitated ad-hoc meetings or skipped meetings. The regular meeting cadence fostered a sense of purpose, increased collaboration, allowed for research projects better understanding each other, and ultimately increased alignment and support for RISE CDEs. This is critical. In our experience, this should involve an experienced facilitator who is flexible and is focused on efficiency and minimizing partner and community burden. Although virtual meetings were costly in terms of time, they fostered a higher level of commitment from the research team members. Virtual meetings facilitated discussion and an understanding of the values and motivations behind the ratings and feedback, particularly given the diversity of project interventions and geographic locations. Balancing both virtual meetings and electronic feedback was essential for efficiency and addressing nuanced feedback. This fostered development of CDEs that are relevant to a wider audience, enhancing their utility and effectiveness.

Flexibility in meeting cadence and engagement was necessary for the research projects and Coordinating Center throughout the phases of the data harmonization process. Research projects had differing levels of engagement, influenced by bandwidth and time constraints. The Coordinating Center adopted a strategy of summarizing feedback and did not require 100% response rates to progress to the next iteration; in instances where input was missing or incomplete, the Coordinating Center used voting and feedback from prior iterations to maintain momentum while having each iteration build on the last.

### Flexible strategy for adapting to diverse project needs

Two key aspects of the prospective harmonization process were maintaining flexibility and deciding clear points of action when partner sites suggested or required changes or customizations. When significant deviations emerged from a particular project, we followed several action steps to reach a resolution, depending on the needs of the site and proposed changes to the CDE(s). There were four potential courses of action in these circumstances: expert panel discussion, project-specific consideration, exemptions, or retaining the CDE. This included expert panel discussion; project specific consideration, project specific exemption and retention of CDE. If the changes to a CDE suggested by a partner could potentially benefit all projects, the suggestions were taken to the full group for consideration. In some cases, the Coordinating Center requested feedback in documents and survey questions; in other cases, the Coordinating Center brought the topic to a virtual discussion for live feedback. If the suggested change was deemed necessary for the one project to include but not reasonably applicable to the full group, then project-specific changes to the survey were allowed. In these cases, if the needed changes did not subtract from the common survey’s purpose, that one project would modify their CDE, without requiring prospective harmonization.

In other cases, where the changes requested were more significant, but deemed necessary, project-specific exemptions were made. In these cases, the project was customized to the local context while preserving core CDE intent. In these cases where items are not identical, retrospective harmonization would be an option. Additionally, some instances had significant changes suggested, but none of the prior three options were feasible, so the project was encouraged to adopt the original CDE. Overall, this approach reflects a deliberate balance where flexibility was not compromised but intentional allowing measures to remain responsive to local context while preserving conceptual alignment across studies. In dynamic and evolving areas like harm reduction, CDEs must be both stable and nimble [14, 16, 18–20]. We would also like to note that the strategy for managing variation was not based on enforcement but on shared decision making and flexibility. Research projects collaborated with the Coordinating Center to identify modifications that preserved the integrity of the shared framework while meeting site-specific needs. This approach recognized the varied realities of each project anchored in mutual goals rather than mandated conformity.

## Results

### Mapping finalized project measures with CDEs

As we received the finalized surveys from research projects, we began to map each questionnaire to the finalized set of CDEs. This analysis was conducted on questionnaires as each was submitted to the Coordinating Center, so each site could be mapped individually. First, we conducted a general assessment of the submitted survey to broadly map each measure to Tier 1 or Tiers 2 or 3. This was done under general terms only as we were not looking for exact matches to data elements. We used a keyword search based on terms from the survey question to identify similar measures in either Tier 1 or Tiers 2 and 3 to find at least one CDE that resembled the measure. Each measure was then color-coded to reflect whether it fell under Tier 1, Tiers 2 or 3, or neither (i.e., that it was an entirely new construct from the research site). Table 3 includes the Tier 1 CDE and outcomes.

Once this initial assessment was completed, the survey was mapped to the CDEs. A system was created in a spreadsheet to track the mapping process, and all measures (including those captured by electronic health records) submitted by each site were added to the spreadsheet. Each measure from the Tier 1 and Tiers 2 or 3 CDEs occupied an individual row, and one column per survey was added to track changes made to each measure at the site level. Each survey was reviewed by one researcher. First, the Tier 1 measures (identified in the initial assessment process) were compared to Tier 1 CDEs. Based on the wording of the question, the identical or most similar measure was identified. Depending on whether the measure was modified from the original CDE and in what way, it was categorized into the appropriate color category in the tracking spreadsheet: identical question and options; modified question, identical response options; identical question, modified response options; both question and response options are modified; similar question included, but likely too different to compare; and measure not included. In cases where the CDE was included as a measure but was modified, comments were added to the spreadsheet to provide detail on how the measure had been modified. The modified wording was also included in the spreadsheet.

**Fig. 2:**
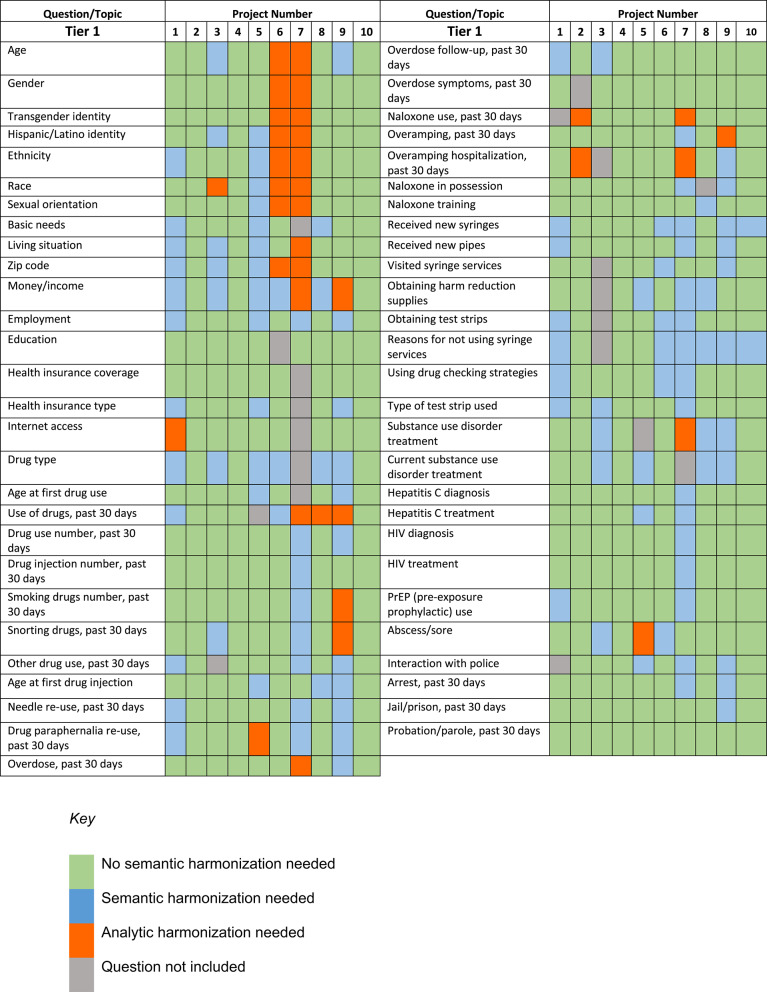
Heat Map of Tier 1 Coverage and Harmonization Needs for CDEs and COMs

This mapping process was completed for all surveys across all 10 Research Projects. We found that, on average, 74% of included Tier 1 CDEs were identical to the original, 5% of included Tier 1 CDEs were modified in question stem only, 12% of included Tier 1 CDEs were modified in response option only, and 5% of included Tier 1 CDEs were modified in both response option and question. Of the Tier 1 CDEs that were included, an additional 4% were modified too significantly to allow for data integration without need for analytic harmonization. Taken together, this resulted in about 94% coverage on average of Tier 1 measures and 100% coverage of common outcome measures (COMs). This allowed research projects to contribute CDEs and COMs but also retain nuances of their individual study assessment battery. See Fig. 2 for a heat map of Tier 1 measure coverage and harmonization needs.

During the prospective harmonization process, as the CDEs were being developed, the methods used had to evolve over each iteration. This was needed to ensure that each new iteration of the process, particularly the methods used to collect site feedback, was appropriate for the type of data being collected and responsive to the RISE research and community members’ needs. Over the iterations, we varied between collecting quantitative or qualitative feedback and in some cases collected both simultaneously. Quantitative feedback allowed us to more clearly categorize individual measures based on their scores, which made it feasible to create clear guidelines on when a measure would remain in Tier 1 or be considered for Tiers 2 or 3. Quantitative scoring also could quickly identify measures for which there was disagreement among research projects, those measures with a large range of scores could be pulled out for further analysis and feedback, to determine why there were discrepancies in scoring between research projects. In those cases, where additional details were needed to determine a path forward, qualitative feedback was essential. In several iterations of the CDEs, qualitative feedback was requested from research projects to get deeper and more nuanced comments. Qualitative feedback also provided us with a better understanding of the perspectives of each site as a whole and revealed some of the variations in priorities between research projects, depending on the populations they served.

Though we primarily elicited feedback through these documents, we also made use of feedback in later iterations, during facilitated virtual discussion. This was necessary in a later iteration to clarify ratings of measures where voting had been unclear, feedback had been conflicting, or qualitative feedback had been inconclusive. All submitted feedback on these measures was compiled into a slide deck and presented for discussion with the research projects. These live virtual meetings required a large input of time and commitment from research projects, but they generated a high level of buy-in and investment from the researchers. These virtual settings helped the RISE Network clarify its values and motivations and helped reveal the motivations and priorities of individual research projects. Though it was not feasible to collect feedback in this live format for all iterations, including the virtual component alongside digital feedback helped increase buy-in from the research projects and brought nuance to the measures being developed.

By conducting an extensive prospective harmonization process, we developed nuanced and thorough CDEs. This process also achieved a high level of buy-in from all RISE Network research projects for using these measures. Consequently, when the research projects developed their individual surveys, there was significant overlap with the Tier 1 CDEs. While the prospective harmonization process was time-intensive and labor-intensive, it ensured that subsequent harmonization efforts were primarily semantic rather than analytic. Semantic harmonization, which does not require specialized software or analytic skills, is much easier to achieve. Thus, the upfront investment in prospective harmonization significantly reduced the need for retrospective analytic harmonization of the final site surveys.

### Effort and engagement required for prospective harmonization

As discussed above, the prospective harmonization process was both resource and labor intensive. Over a 9- month period, the RISE Network Coordinating Center facilitated 12 virtual meetings, including monthly calls each with full Network participation and additional ad hoc calls to address specific measures. We had full participation from the RPs in each formal Delphi iteration and all contributed structured feedback through both qualitative and quantitative formats. Community input was solicited during multiple phases through established advisory boards and partner organizations. Feedback from each site was tracked by measure and over 300 individual comments were reviewed and synthesized across the 8 formal iterations. In total over 100 measures were proposed, evaluated and refined with their inputs, with final Tier 1 inclusion reflecting consensus on both feasibility and scientific relevance from Delphi process. The level of structured collaboration required significant investment of time and coordination but resulted in a high degree of project alignment and measure uptake. As mentioned above, the development and implementation of CDEs was a funded activity required both research projects and the Coordinating Center as part of the scope of their awards. While this process is labor intensive, the high degree of alignment achieved across projects demonstrates the value of investing in prospective harmonization to support cumulative, scalable evidence generation relative to retrospective alignment efforts of this caliber for data integration.

## Discussion

This paper outlines the process of harmonizing CDEs and COMs to standardize data collection and reporting for harm reduction research. We detail the results of a community-informed, consensus-driven approach implemented by the NIDA HEAL RISE Network and highlight key lessons learned from this prospective harmonization process. These insights can inform others seeking to construct CDEs and COMs using similar methodologies.

### Challenges in prospective data harmonization

One significant challenge was the variation in terminology and definitions used across different studies. Each site provided input on how terminology should be used and in what context, informed by their specific populations and prior survey experiences. To address this, we developed a comprehensive glossary of terms that provided clear and standardized definitions for key concepts. Research projects also needed to adapt the instruments to their study sites, considering factors such as cultural differences and local regulations. Regular meetings, facilitated discussions, and opportunities for both quantitative and qualitative feedback allowed the research teams and the harmonization committee to reach a consensus on these challenges. In cases where adaptations were necessary, they were generally minor due to the thoroughness of the prospective harmonization process, thereby minimizing the need for retrospective analytic harmonization. Additional challenges included individual project pressures for data collection startup. Despite our efforts in prospective harmonization, some level of retrospective harmonization will inevitably be required for broad Network analysis, given that the use of common measures was not globally aligned. However, these instances are expected to be minimal and do not detract from the overall strengths of the initiative.

### Lessons learned

The process of identifying and implementing Tier 1 items was not without challenges. Regular meetings and discussions between research teams and the harmonization committee facilitated the resolution of these issues. However, this process requires significant time for iterations, thoughtful communication, and buy-in from participating research projects. Because the research projects were extensively involved in developing the measures, they were highly committed to using them once finalized. The iterative process which included quantitative, qualitative, and live feedback from research projects ensured that each site had ample opportunity to contribute to the development of CDEs, enhancing their relevance to specific research areas. This level of prospective detail significantly reduced the need for retrospective analytic harmonization, which would otherwise require considerable time, resources, and expertise [21, 22]. Additionally, given that this was a time-consuming endeavor, it was important center the development of CDEs on the need for integrated data analyses, therefore the RISE Network expert panel members, each representing a research project, had to collectively agree to a pre-determined list of analytical aims as an important rationale for prospective harmonization.

The process highlights the Coordinating Center’s role in facilitating these activities. While individual research projects focused on getting their study-specific objectives underway, the Center compiled Network feedback and made necessary adjustments. This flexible yet resource-intensive work required significant time and effort but was critical for successful harmonization. To support broader uptake and application, Tier 1 CDEs and COMS developed by the RISE Network will be made publicly available on NIDA websites. In addition, the Coordinating Center has developed 508-compliant interactive PDFs of all measures and is disseminating these resources through conference presentations, peer networks and technical assistance efforts. These dissemination strategies are designed to increase accessibility for researchers, public health departments, and harm reduction programs [18].

### Broader impacts

The adoption of community-informed harm reduction CDEs and COMs offers several significant benefits, including improved standardization, enhanced data quality, and increased collaboration and efficiency. The flexibility of these CDEs and COMs ensures adaptability to the evolving harm reduction landscape, maintaining the relevance and impact of research. By incorporating new knowledge and innovations, CDEs and COMs play a pivotal role in advancing harm reduction efforts nationally. The broad use of standardized CDEs and COMs enhances the quality and impact of research and ensures that findings effectively serve the diverse communities and individuals most in need of harm reduction interventions. Given the need to expand the evidence base in support of harm reduction strategies, having CDEs and COMs implemented across studies facilitates cumulative science, which can accelerate the development of evidence for various harm reduction strategies. This prospective approach is well-suited for other areas of substance use research where interventions and contexts are changing rapidly such as cannabis, synthetic opioids, or stimulant use. By emphasizing community-informed adaptation and semantic flexibility, the RISE Networkmodel allows CDEs to remain responsive while maintaining sufficient consistency for pooled analyses. As new products enter the marketplace and patterns of use evolve, availability of CDEs and prospective community informed harmonization strategies like this can help researchers stay aligned with real world practice and community needs [23, 24].

Although the RISE Network is still in early stages of study implementation, the foundational work of prospective harmonization has already resulted in alignment across all studies. All research projects adopted the majority of Tier 1 CDEs with minimal revision and most of the COMs (See Fig. 2). While cross-site analyses and synthesis are still forthcoming, these early markers of alignment lay the groundwork for more efficient, coordinated, and scalable evidence generation. This collaborative infrastructure represents an important step towards continued building of a cumulative community-informed science of harm reduction [25]. By strengthening the infrastructure for shared learning, we create space for more coordinated and effective strategies that reflect the insights and resilience of the communities we serve.

**Table 3:** Constructs Comprising Tier 1 Common Data Elements (CDEs) and Common Outcome Measures (COMs).

Tier 1 CDEs
Sociodemographic characteristics
Drug use history
Overdose experiences
Harm reduction services access and utilization
Substance use disorder treatment
Hepatitis C or HIV treatment
Skin and soft tissue infection occurrence
Criminal legal system involvement

Tier 1 COMs
Harm reduction service utilization
Opioid use disorder or substance use disorder treatment (including prescriptions)
Drug use behaviors
Overdose
Social determinants of health

## Data Availability

Not applicable.
